# Does knowledge of blood calcium concentration at 2 days postpartum affect decisions of calcium supplementation?

**DOI:** 10.3168/jdsc.2023-0456

**Published:** 2023-11-17

**Authors:** H.A. McCray, C.R. Seely, J.A.A. McArt

**Affiliations:** 1 College of Veterinary Medicine, Cornell University, Ithaca, NY 14853; 2 Department of Population Medicine and Diagnostic Sciences, College of Veterinary Medicine, Cornell University, Ithaca, NY 14853

## Abstract

Delaying oral Ca supplementation might benefit cows with low blood Ca concentrations at 4 d in milk (DIM), a time when reduced blood total Ca (tCa) is associated with negative health and production outcomes. To implement a targeted approach to manage subclinical hypocalcemia (SCH) at the herd level, it is important to identify which cows benefit from supplemental Ca. Therefore, our objective was to determine if SCH diagnosis at 2 DIM could inform decisions of oral Ca supplementation at 2 and 3 DIM based on milk yield and 4 DIM blood Ca concentration. Data were analyzed from a previously conducted randomized controlled trial on multiparous cows (n = 518) from 4 farms in New York State. Cows were randomly assigned to 1 of 2 treatment groups at calving: (1) control (CON; no Ca supplementation, n = 259) or (2) bolus (BOL; 43 g of oral Ca administered at 2 and 3 DIM postcalving, n = 259). For each parity group (2, 3, 4+), we used generalized linear mixed models to identify serum tCa concentrations at 2 DIM that maximized the difference in milk yield to diagnose SCH. Cows were classified as normocalcemic (NC; parity 2 tCa >1.9 mmol/L, parity 3 tCa >1.87 mmol/L, n = 327; parity ≥4 had no defining threshold) or SCH (parity 2 tCa ≤1.9 mmol/L, parity 3 tCa ≤1.87 mmol/L, n = 58; parity ≥4 had no defining threshold). Parity 2 and 3 cows were further classified into 1 of 4 SCH-treatment groups (SCHTRT) based on 2 DIM SCH status and random treatment allocation: (1) NC-CON, n = 165, (2) SCH-CON, n = 28, (3) NC-BOL, n = 162, or (4) SCH-BOL, n = 30. Generalized linear mixed models were used to analyze the difference in milk yield for the first 10 wk of lactation and tCa at 4 DIM between SCHTRT groups with separate analyses performed for parities 2 and 3. Mean milk yield differed between SCHTRT groups for both parities. For parity 2, SCH-CON and SCH-BOL cows produced more milk than NC-CON and NC-BOL cows with SCH-CON producing 50.9 (95% confidence interval [CI] = 48.4, 53.4) kg/d, SCH-BOL 51.7 (49.1, 54.2) kg/d, NC-CON 47.5 (46.3, 48.7) kg/d, and NC-BOL 47.2 (45.8, 48.5) kg/d of milk. Milk yield was also different between SCHTRT groups for parity 3 with SCH-BOL cows producing more milk than NC-CON and NC-BOL cows. In parity 3, SCH-BOL cows produced 56.3 (95% CI = 53.1, 59.3) kg/d, SCH-CON 51.7 (48.6, 54.7) kg/d, NC-BOL 50.6 (49.0, 52.2) kg/d, and NC-CON 48.7 (46.9, 50.5) kg/d of milk. For both parities, SCH-CON and SCH-BOL cows had lower tCa at 2 DIM than NC-CON and NC-BOL cows. At 4 DIM, tCa concentrations were similar for all SCHTRT groups respective to parity. Our results suggest that although delayed Ca bolus administration does not improve blood Ca concentration when compared with controls, it does support increased milk production in parity 3 cows regardless of Ca status at 2 DIM. Thus, knowledge of blood Ca at 2 DIM should not affect decisions of Ca supplementation in this parity of cows.

## Graphical Abstract

**Figure:**
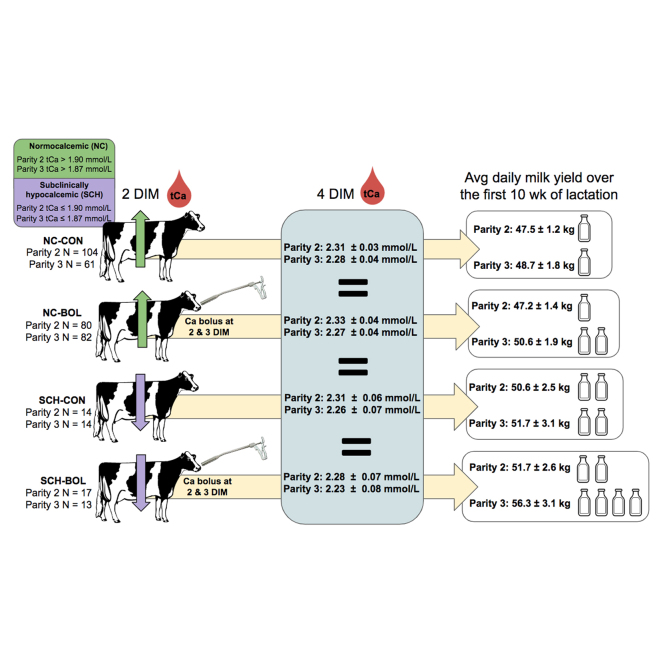

The transition from pregnancy to lactation for the modern dairy cow is an extreme metabolic challenge due to the dramatic increase in nutrient and macromineral demands (Goff et al., 2002). Consequently, health challenges during the periparturient period are not uncommon, some of which are subclinical in nature. Of note, approximately 50% of multiparous dairy cows experience some form of subclinical hypocalcemia (**SCH**; Reinhardt et al., 2011; McArt and Neves 2020) as the start of milk production almost doubles the cow's Ca requirement (Goff et al., 2002). Although this decrease in blood Ca is not accompanied by any physical manifestations of disease and must be diagnosed by measurement of blood Ca, SCH diagnosed after 1 DIM is reliably associated with negative health and production outcomes (Caixeta et al., 2017; Neves et al., 2018; Venjakob et al., 2018). Specifically, SCH occurring at or persisting through 4 DIM, newly coined as dyscalcemia, is more commonly associated with decreased intake and milk production, increased risk for adverse health events, and reduced reproductive success, whereas transient reductions in blood Ca that do not extend beyond 1 DIM are associated with improved production and cow health (McArt and Neves, 2020; Seely et al., 2021; Seely and McArt, 2023).

Oral Ca supplementation, most commonly administered in the form of a bolus at calving and 24 h later, has been used in a blanket fashion with the goal of improving blood Ca concentrations and reducing adverse health events associated with SCH; however, several studies have shown the efficacy of oral Ca to vary widely, and thus their use at the conventional time after calving can be debated (Valldecabres et al., 2023). Recent work by Seely et al. (2024) assessed if delaying oral Ca supplementation to 2 and 3 DIM, from traditionally administered times at calving and at 1 DIM, would reduce the incidence of low Ca concentrations at 4 DIM and ameliorate the associated reductions in milk yield found in dyscalcemic cows. They reported a limited impact of herd-level delayed supplementation on blood Ca concentrations at 4 DIM, although milk yield was increased in parity 3 cows. However, it remains unanswered if cows with SCH at 2 DIM have differing benefits from delayed Ca supplementation than normocalcemic (**NC**) cows at 2 DIM.

Therefore, the objective of our study was to determine if SCH diagnosis at 2 DIM could inform decisions of oral Ca supplementation at 2 and 3 DIM based on milk yield and 4 DIM blood Ca concentration. We hypothesized that cows with SCH receiving an oral bolus at 2 and 3 DIM would have improved milk yield and blood Ca concentration at 4 DIM when compared with SCH controls.

Our animal use protocol was approved by the Cornell University Institutional Animal Care and Use Committee, protocol number 2020–0102. Our analysis was conducted on data previously collected from a prospective, randomized control trial as described by Seely et al. (2024) and was written following the REFLECT statement (Sargeant et al., 2010).

Data were collected from 4 farms in northeastern (farms A and B) and central (farms C and D) New York State. Farms were selected based on the following criteria: feed a TMR and prepartum negative DCAD diet, milk at least 1,000 Holstein cows 3 times a day, record daily milk weights using DairyComp 305 (Valley Agricultural Software, Tulare, CA), have headlocks in the fresh cow pens, and allow the research team access to computer records for 6 mo after the enrollment of the last cow. Data were collected from June 2 to July 10, 2021, on farms A and B and from July 15 to September 25, 2021, on farms C and D. All multiparous cows that calved during the sampling period were eligible for enrollment. At calving, farm personnel enrolled cows using a premade treatment allocation log that randomly assigned cows to 1 of 3 treatment groups: (1) cows receiving no Ca supplementation (**CON**; n = 305); (2) cows receiving a 43-g oral Ca bolus containing CaCl_2_ and CaSO_4_ (Bovikalc bolus, Boehringer Ingelheim, St. Joseph, MO) administered at calving and 1 DIM, or (3) cows receiving an oral Ca bolus (Bovikalc bolus) administered at 2 and 3 DIM (**BOL**; n = 299). Data were analyzed only from CON and BOL groups for cows with a previous lactation gestation length >260 d, cows which did not receive exogenous Ca <4 DIM, cows which remained in the herd ≥4 DIM, and cows with blood samples collected at 2 and 4 DIM, which provided a total of 518 cows for analysis (farm A, n = 129; farm B, n = 55; farm C, n = 245; farm D, n = 89).

Sample collection and treatment administration occurred at the same time each day on all farms but differed in their relation to fresh feed delivery (farm A: blood sample at 0500 h, feed delivery at 0600 h; farm B: blood sample at 1100 h, feed delivery at 1000 h; farm C: 0700 h, feed delivery at 0800 h; farm D: blood sample at 0900 h, feed delivery at 0800 h). Blood samples were collected before treatment administration at 2 and 4 DIM from coccygeal vessels using a 20-G Vacutainer needle (Becton Dickinson, Franklin Lakes, NJ) and a 10-mL evacuated tube with no anticoagulant (Becton Dickinson) and immediately placed on ice. Following blood sampling, the treatment team administered boluses to cows that were 2 and 3 d postpartum. Blood samples were transported to the laboratory for processing and centrifuged for 20 min at 2,000 × *g* at 15°C within 2 h of collection. Serum was then aliquoted into 1.7-mL microfuge tubes and stored at −20°C until analysis. Serum samples were analyzed for tCa using commercially available kits (Calcium Gen.2, Roche Diagnostics, Indianapolis, IN) at the New York State Animal Health and Diagnostic Center (Ithaca, NY) on an automated analyzer (Hitachi Modular P800, Roche Diagnostics). Inter- and intra-assay coefficients of variation were 1.1% and 0.9%, respectively.

To diagnose SCH, for each parity group (2, 3, ≥4) we first identified the interquartile range of tCa concentrations and divided that range into 11 cutpoints in 0.02 or 0.03 mmol/L intervals. We then created generalized linear mixed models through the MIXED procedure of SAS (v.9.4, SAS Institute Inc.) to determine milk production differences between cows above or below the analyzed tCa cutpoint. Models included the fixed effects of farm, week of lactation, 2 DIM Ca status, and the interaction of week × 2 DIM Ca status. The final tCa cutpoint for each parity group was chosen based on the model that maximized the difference in milk production between cows above or below the cutpoint where cows below the cutpoint, deemed SCH, produced more milk than those above the cutpoint (Table 1). This definition of diagnosis was chosen as cows with a transient reduction in tCa at 1 DIM produce more milk throughout lactation compared with cows with normal tCa at 1 DIM (Neves et al., 2018; McArt and Neves, 2020; Seely et al., 2021). For parity 2, milk production differences were maximized at a tCa cutpoint of 1.90 mmol/L (normocalcemia [**NC**], n = 184; SCH, n = 31), and for parity 3, milk production differences were maximized at a tCa cutpoint of 1.87 mmol/L (NC, n = 143; SCH, n = 27). There was no tCa cutpoint that yielded a difference in milk production for parity ≥4 cows, and thus parity ≥4 were excluded from further analysis (Table 1). Parity 2 and 3 cows were then further classified into 1 of 4 SCH-treatment groups (**SCHTRT**) based on their 2 DIM SCH status and random treatment allocation: (1) **NC-CON** (parity 2, n = 104; parity 3, n = 61), (2) **SCH-CON** (parity 2, n = 14; parity 3, n = 14), (3) **NC-BOL** (parity 2, n = 80; parity 3, n = 82), or (4) **SCH-BOL** (parity 2, n = 17; parity 3, n = 13).

**Table 1:** Modeled mean milk yield for the first 10 wk of lactation at different serum total Ca (tCa) cutpoints at 2 DIM in multiparous Holstein cows (n = 518) from 4 farms in New York State^1^

tCa cutpoint, mmol/L	% (n) at or below cutpoint	% (n) above cutpoint	Milk yield above cutpoint, kg (95% CI)	Milk yield at or below cutpoint, kg (95% CI)	*P*-value
Parity 2					
1.5	0 (1)	100 (214)	—	—	—
1.75	5 (10)	95 (205)	47.3 (45.8, 48.7)	51.7 (45.4, 57.9)	0.2
1.8	8 (17)	92 (198)	47.1 (45.7, 48.6)	51.4 (46.5, 56.3)	0.1
1.85	10 (22)	90 (193)	47.2 (45.7, 48.7)	49.8 (45.6, 54.0)	0.2
1.87	10 (22)	90 (193)	47.2 (45.7, 48.7)	49.8 (45.6, 54.0)	0.2
1.9	14 (31)	86 (184)	46.9 (45.4, 48.4)	51.3 (47.7, 54.8)	0.02
1.92	14 (31)	86 (184)	46.9 (45.4, 48.4)	51.3 (47.7, 54.8)	0.02
1.95	19 (41)	81 (174)	46.6 (45.1, 48.1)	51.0 (48.1, 53.9)	0.009
2	29 (62)	71 (153)	46.7 (45.0, 48.4)	49.3 (46.9, 51.7)	0.07
2.25	78 (167)	22 (48)	46.6 (43.9, 49.4)	47.8 (46.2, 49.3)	0.4
2.5	99 (213)	1 (2)	44.6 (32.6, 56.6)	47.6 (46.2, 49.0)	0.6
Parity 3					
1.5	1 (2)	99 (168)	48.4 (46.3, 50.5)	44.4 (28.3, 60.4)	0.6
1.75	10 (17)	90 (153)	47.9 (45.6, 50.1)	50.1 (45.4, 54.7)	0.4
1.8	14 (24)	86 (146)	47.7 (45.4, 49.9)	50.5 (46.1, 54.6)	0.2
1.85	16 (27)	84 (143)	47.3 (45.0, 49.6)	51.2 (47.3, 55.0)	0.08
1.87	16 (27)	84 (143)	47.3 (45.0, 49.6)	51.2 (47.3, 55.0)	0.08
1.9	19 (33)	81 (137)	47.7 (45.2, 50.2)	49.3 (45.9, 52.8)	0.4
1.92	19 (33)	81 (137)	47.7 (45.2, 50.2)	49.3 (45.9, 52.8)	0.4
1.95	25 (42)	75 (128)	48.4 (45.8, 51.0)	48.1 (45.0, 51.1)	0.9
2	32 (54)	68 (116)	49.5 (46.8, 52.3)	46.9 (44.2, 49.7)	0.2
2.25	81 (137)	19 (33)	50.9 (45.8, 56.1)	48.0 (45.9, 50.1)	0.3
2.5	100 (170)	0 (0)	—	—	—
Parity ≥4					
1.5	5 (7)	95 (126)	46.2 (43.5, 48.9)	45.9 (36.0, 55.7)	1
1.75	22 (29)	78 (104)	46.2 (43.1, 49.2)	46.2 (41.7, 50.7)	1
1.8	26 (35)	74 (98)	46.0 (42.9, 49.1)	46.7 (42.4, 51.0)	0.8
1.85	35 (47)	65 (86)	46.5 (43.3, 49.7)	45.8 (42.0, 49.6)	0.8
1.87	35 (47)	65 (86)	46.5 (43.3, 49.7)	45.8 (42.0, 49.6)	0.8
1.9	41 (55)	59 (78)	46.9 (43.7, 50.2)	45.2 (41.7, 48.8)	0.4
1.92	41 (55)	59 (78)	46.9 (43.7, 50.2)	45.2 (41.7, 48.8)	0.4
1.95	49 (65)	51 (68)	46.9 (43.6, 50.3)	45.3 (41.8, 48.8)	0.5
2	56 (75)	44 (58)	46.2 (42.5, 50.0)	46.1 (42.9, 49.3)	1
2.25	86 (114)	14 (19)	43.3 (37.8, 48.8)	46.7 (44.0, 49.5)	0.2
2.5	100 (133)	0 (0)	—	—	—

1 Eleven cutpoints in 0.02 or 0.03 mmol/L intervals were identified based on the interquartile range of tCa concentrations. Models were created separately for parity 2 (n = 215), parity 3 (n = 170), and parity ≥4 (n = 133) cows.

We did not calculate an a priori sample size as our study was based on data from Seely et al. (2024). Comparing differences in mean milk yield between CON and BOL groups within parity group (2, 3), we conducted a post hoc power analysis using ClinCalc (ClinCalc LLC, https://clincalc.com). For parity 2, mean (± SD) milk yield of SCH cows was 50.9 ± 11.4 kg/d (CON) and 51.7 ± 11.6 kg/d (BOL) and mean (± SD) of NC cows was 47.5 ± 7.7 kg/d (CON) and 47.2 ± 8.1 kg/d (BOL); accounting for a type I error of 5%, our available sample size provided us with a 4% power to assess differences in milk yield following CON and BOL treatments between both SCH and NC groups. Similar calculations for parity 3 SCH cows were mean (± SD) milk yields of 51.8 ± 12.6 kg/d (CON) and 56.3 ± 13.3 kg/d (BOL) and mean (± SD) of NC cows of 48.7 ± 9.5 kg/d (CON) and 50.6 ± 9.0 kg/d (BOL); accounting for a type I error of 5%, our available sample size provided us with a 15% and 23% power for milk yield outcomes between SCH and NC groups, respectively.

For each parity group, generalized linear mixed models were used to analyze the difference in milk yield for the first 10 wk of lactation and tCa at 2 and 4 DIM among SCHTRT groups using the MIXED procedure of SAS. For the milk yield model, multiple measurements over time were analyzed using the repeated statement and included the random effect of cow and fixed effects of farm, week, SCHTRT, and their respective 2-way interactions. Variables and interactions were removed in a backward stepwise fashion if *P* > 0.1. Regardless of the *P*-value, the fixed effects of farm, week, SCHTRT, and SCHTRT × week remained in the final model. The tCa model included the fixed effects of SCHTRT and farm. An autoregressive (1) covariance structure was used in all models. When main effects and least squares means were different between groups, Tukey-Kramer studentized adjustments were used to account for multiple comparisons. To improve the normality of the residuals, 2 DIM tCa was log transformed for parity 2 and square root transformed for parity 3; 4 DIM tCa and milk yield were squared for both parity models to improve the normality of the residuals. Means presented hereafter represent back-transformed means and their associated 95% confidence intervals (**CI**).

Milk yield differed by parity group with parity 3 cows producing more milk than parity 2 cows. Milk yield also differed by week (*P* < 0.001) for both parity 2 and 3 cows with peak milk yield occurring at 8 and 6 wk, respectively, when controlling for farm (*P* < 0.001). Mean milk yield differed between SCHTRT groups for both parities (Figure 1). For parity 2, SCH-CON and SCH-BOL cows produced more milk than NC-CON and NC-BOL cows (all *P* < 0.08). Parity 2 SCH-CON produced 50.9 (95% CI = 48.4, 53.4) kg/d, SCH-BOL 51.7 (49.1, 54.2) kg/d, NC-CON 47.5 (46.3, 48.7) kg/d, and NC-BOL 47.2 (45.8, 48.5) kg/d of milk. Milk yield was also different between SCHTRT groups for parity 3 (*P* = 0.001) with SCH-BOL cows producing more milk than NC-CON and NC-BOL cows (*P* < 0.001 and 0.01, respectively). Parity 3 SCH-BOL cows produced 56.3 (95% CI = 53.1, 59.3) kg/d, SCH-CON 51.7 (48.6, 54.7) kg/d, NC-BOL 50.6 (49.0, 52.2) kg/d, and NC-CON 48.7 (46.9, 50.5) kg/d of milk.

**Figure 1:**
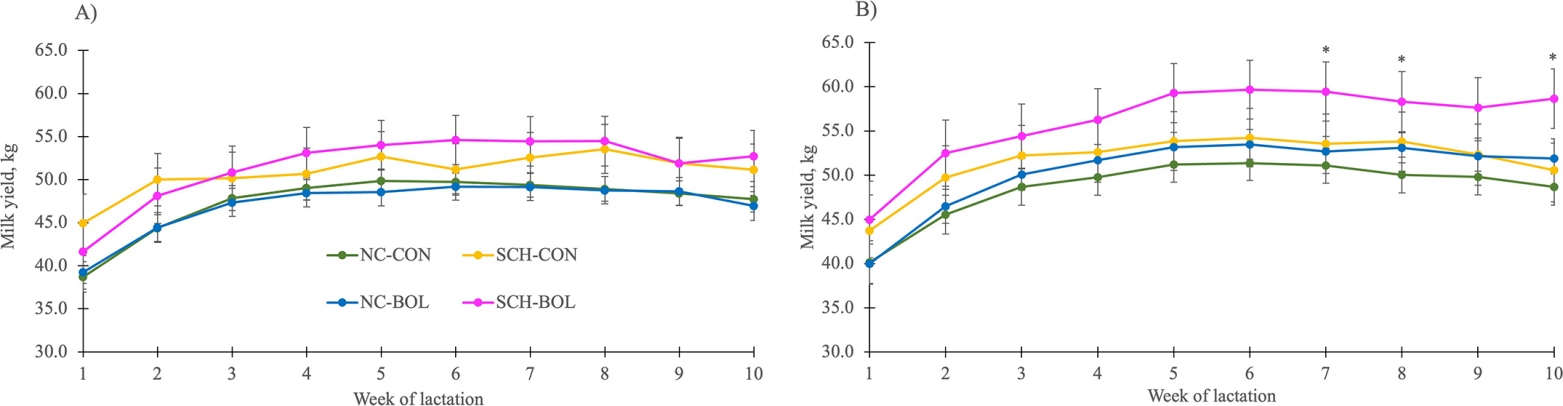
Modeled means of average weekly milk yield for parity 2 (A; n = 215) and 3 (B; n = 170) Holstein cows from 4 farms in New York State categorized by 2 DIM serum total Ca (tCa) status and random treatment allocation: (1) normocalcemic cows (NC; parity 2 tCa >1.9 mmol/L, parity 3 tCa >1.87) receiving no supplemental Ca (NC-CON; parity 2, n = 104; parity 3, n = 61), (2) subclinically hypocalcemic cows (SCH; parity 2 tCa ≤1.9, parity 3 tCa ≤1.87) receiving no supplemental Ca (SCH-CON; parity 2, n = 14; parity 3, n = 14), (3) NC cows receiving an oral Ca bolus (43 g of Ca) at 48 and 72 h postcalving (NC-BOL; parity 2, n = 80; parity 3, n = 82), or (4) SCH cows receiving an oral Ca bolus (43 g of Ca) at 48 and 72 h postcalving (SCH-BOL; parity 2, n = 17; parity 3, n = 13). Asterisks denote differences between NC-CON and SCH-BOL, where *P* < 0.05. Error bars represent 95% CI.

We did not find a difference in milk yield of parity 2 cows within NC or SCH groups following oral Ca bolus administration at 2 and 3 DIM. Given the underpowering of our study, we are unable to conclude if delayed Ca bolus administration truly provided no support for an increase in milk yield, and further studies are needed to understand if our results represent a type II error. Alternatively, this lack of difference might be explained by lower milk production in parity 2 cows without as large of a demand for Ca by the mammary gland compared with parity 3 cows. Considering our results, it might be unnecessary to provide parity 2 cows with supplemental Ca at 2 and 3 DIM, and further studies should assess if bolusing should be reserved for more mature, higher producing animals.

Parity 3 NC-BOL cows produced 1.9 kg/d more milk over the first 10 wk of lactation than NC-CON cows, and SCH-BOL cows produced 4.6 kg/d more milk than SCH-CON cows of the same parity. These results suggest that regardless of Ca status at 2 DIM, parity 3 cows might benefit from oral Ca supplementation at 2 and 3 DIM as both NC-BOL and SCH-BOL cows had improved milk production when compared with controls. Furthermore, SCH-BOL cows produced the most amount of milk, suggesting that 2 and 3 DIM was a beneficial time point to supplement Ca to parity 3 cows with reductions in blood Ca at 2 DIM. Results described by McArt and Neves (2020) suggest that cows with transient reductions in blood Ca produce more milk than dyscalcemic cows. Considering this, along with results from Oetzel and Miller (2012) and Martinez et al. (2016a) who found that providing oral Ca to cows with previous high 305-d mature equivalent milk yield resulted in improved production, it is possible that identifying and providing supplemental Ca to high-producing cows with SCH at 2 DIM could improve Ca homeostasis and help to minimize the risk of cows becoming dyscalcemic.

By design, serum tCa was different at 2 DIM between SCHTRT groups respective to parity groups (Figure 2). For both parities, cows in SCH-CON and SCH-BOL groups had lower tCa at 2 DIM than cows in NC-CON and NC-BOL groups (all *P* < 0.001). The incidence of dyscalcemia (blood tCa ≤2.2 mmol/L at 4 DIM) by SCHTRT for parity 2 cows was 25.0% NC-CON, 18.2% SCH-CON, 20.0% NC-BOL, and 41.7% SCH-BOL cows, and for parity 3 cows was 17.5% NC-CON, 38.9% SCH-CON, 31.3% NC-BOL, and 26.7% SCH-BOL cows. At 4 DIM, tCa concentrations were similar among SCHTRT groups in parity 2 (*P* = 0.7) and 3 (*P* = 0.6), and regardless of SCHTRT, cows in both parity groups had mean tCa ≥2.23 mmol/L.

**Figure 2:**
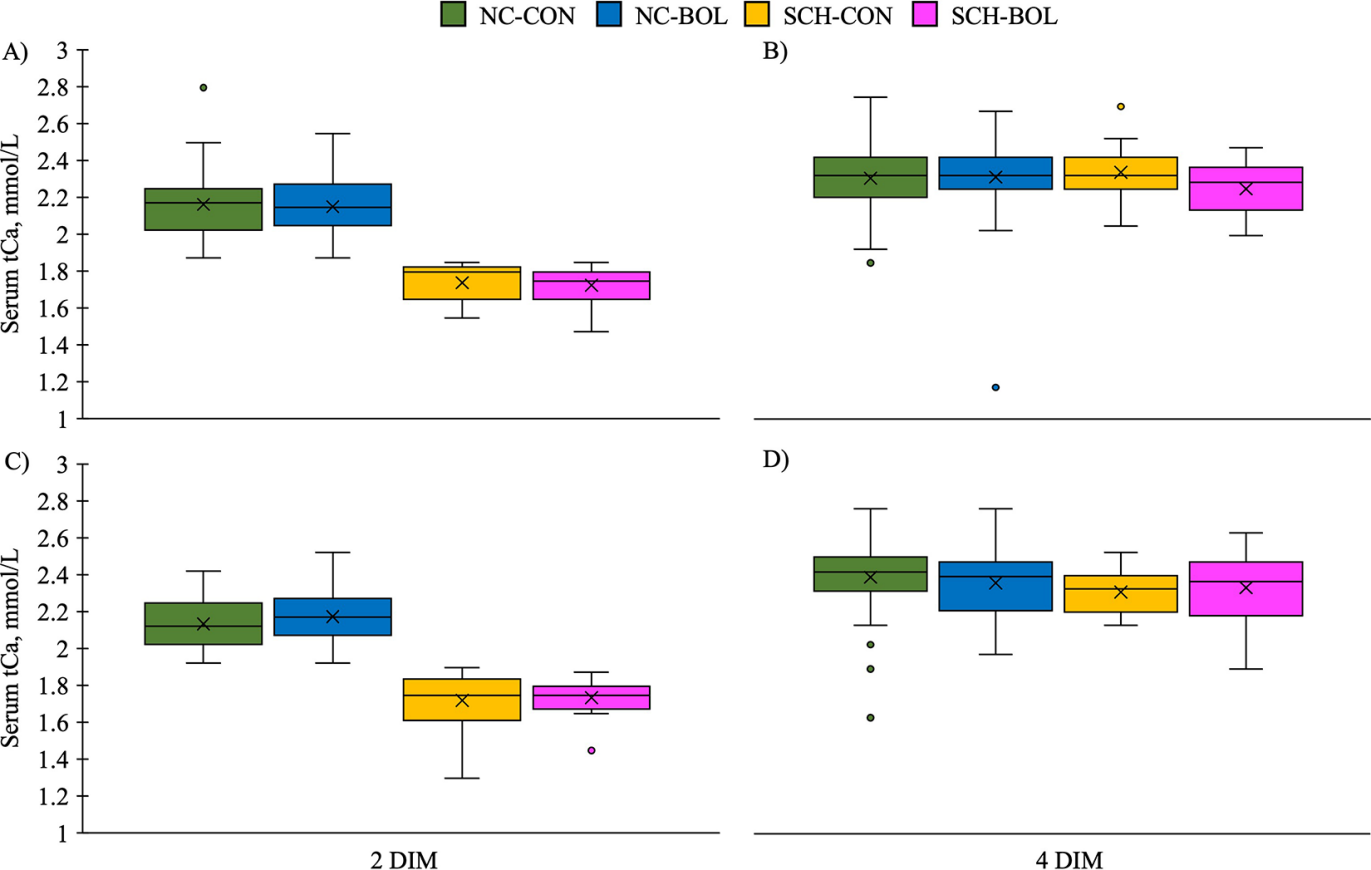
Box and whisker plots of serum total Ca (tCa) for parity 2 (A and B; n = 215) and 3 (C and D; n = 170) Holstein cows from 4 farms in New York State at 2 (A and C) and 4 (B and D) DIM categorized by 2 DIM Ca status and random treatment allocation: (1) normocalcemic cows (NC; parity 2 tCa >1.9 mmol/L, parity 3 tCa >1.87) receiving no supplemental Ca (NC-CON; parity 2, n = 104; parity 3, n = 61), (2) subclinically hypocalcemic cows (SCH; parity 2 tCa ≤1.9, parity 3 tCa ≤1.87), receiving no supplemental Ca (SCH-CON; parity 2, n = 14; parity 3, n = 14), (3) NC cows receiving an oral Ca bolus (43 g of Ca) at 48 and 72 h postcalving (NC-BOL; parity 2, n = 80; parity 3, n = 82), or (4) SCH cows receiving an oral Ca bolus (43 g of Ca) at 48 and 72 h postcalving (SCH-BOL; parity 2, n = 17; parity 3, n = 13). Lower, midline, and upper bounds of boxes refer to 25th, 50th, and 75th percentiles, respectively. Lower and upper whiskers represent minimum and maximums of the interquartile range and individual points represent outliers. Means are indicated by an X.

Despite having lower tCa concentrations at 2 DIM, tCa was similar between all SCHTRT groups by 4 DIM for both parities. This suggests that providing oral Ca supplementation to cows at 2 and 3 DIM has a minimal effect on blood tCa, and cows with SCH at 2 DIM are likely to regain adequate levels of serum tCa regardless of Ca supplementation. Other reports have also shown that the effects of oral bolusing on blood Ca are only transient in nature (Blanc et al., 2014; Domino et al., 2017). When Martinez et al. (2016b) supplemented cows with 1 or 2 oral Ca boluses at calving and 1 DIM, the increase in blood Ca concentrations they observed lasted 2 h and less than 8 h, respectively. Given that we sampled 4 DIM serum tCa approximately 24 h after the 3 DIM boluses were administered, the rise in tCa may not be reflected in our results and was likely already used by the mammary gland for milk production.

Recent studies have shown that low blood Ca at 4 DIM is associated with negative health and production outcomes, and the current strategy of herd-wide Ca supplementation has had minimal success in improving production and cow health. We attempted to determine if diagnosing SCH at 2 DIM and providing oral Ca to this subset of cows would improve milk production and 4 DIM blood tCa concentration. Our results indicate there were minimal effects of delayed bolus administration on milk production in parity 2 cows, suggesting that supplemental Ca might be unnecessary in this younger group of cows; however, further studies with sufficient sample size are needed to fully assess this association. Parity 3 cows benefited from delayed oral Ca supplementation at 2 and 3 DIM regardless of 2 DIM SCH status; therefore, knowledge of blood tCa at 2 DIM should not affect decisions of Ca supplementation in this parity of cows.
